# Efficacy and safety of abiraterone acetate plus prednisolone in patients with early metastatic castration-resistant prostate cancer who failed first-line androgen-deprivation therapy: a single-arm, phase 4 study

**DOI:** 10.1093/jjco/hyaa225

**Published:** 2020-12-15

**Authors:** K Kobayashi, N Okuno, G Arai, H Nakatsu, A Maniwa, N Kamiya, T Satoh, H Kikukawa, Y Nasu, H Uemura, T Nakashima, K Mikami, M Iinuma, K Tanabe, J Furukawa, H Kobayashi

**Affiliations:** Department of Urology, Federation of National Public Service Personnel Mutual Aid Associations Yokosuka Kyosai Hospital, Kanagawa, Japan; Department of Urology, Independent Administrative Institution National Hospital Organization Sagamihara Hospital, Kanagawa, Japan; Department of Urology, Dokkyo Medical University Saitama Medical Center, Saitama, Japan; Department of Urology, Asahi General Hospital, Chiba, Japan; Department of Urology, Independent Administrative Institution National Hospital Organization Shizuoka Medical Center, Shizuoka, Japan; Department of Urology, Toho University Sakura Medical Center, Chiba, Japan; Department of Urology, Kitasato University School of Medicine, Kanagawa, Japan; Department of Urology, Independent Administrative Institution National Hospital Organization Kumamoto Medical Center, Kumamoto, Japan; Department of Urology, Japan Organization of Occupational Health and Safety Okayama Rosai Hospital, Okayama, Japan; Department of Urology and Renal Transplantation, Yokohama City University Medical Center, Kanagawa, Japan; Department of Urology, Ishikawa Prefectural Central Hospital, Ishikawa, Japan; Department of Urology, Chibaken Saiseikai Narashino Hospital, Chiba, Japan; Department of Urology, Independent Administrative Institution National Hospital Organization Mito Medical Center, Ibaraki, Japan; Department of Urology, Tokyo Women’s Medical University Hospital, Tokyo, Japan; Department of Urology, National University Corporation Kobe University Hospital, Hyogo, Japan; Janssen Pharmaceutical K.K., Tokyo, Japan

**Keywords:** abiraterone acetate; chemotherapy-naive metastatic castration-resistant prostate cancer, prednisolone, prostate-specific antigen

## Abstract

**Aim:**

The aim was to evaluate the efficacy and safety of abiraterone acetate plus prednisolone in patients with chemotherapy-naïve early metastatic castration-resistant prostate cancer who failed first-line androgen deprivation therapy.

**Methods:**

Patients with early metastatic castration-resistant prostate cancer with confirmed prostate-specific antigen progression within 1-year or prostate-specific antigen progression without having normal prostate-specific antigen level (<4.0 ng/mL) during first-line androgen deprivation therapy were enrolled and administered abiraterone acetate (1000 mg) plus prednisolone (10 mg). A minimum of 48 patients were required according to Simon’s minimax design. The primary endpoint was prostate-specific antigen response rate (≥50% prostate-specific antigen decline by 12 weeks), secondary endpoints included prostate-specific antigen progression-free survival and overall survival. Safety parameters were also assessed.

**Results:**

For efficacy, 49/50 patients were evaluable. Median age was 73 (range: 55–86) years. The median duration of initial androgen deprivation therapy was 32.4 (range: 13.4–84.1) weeks and 48 patients experienced prostate-specific antigen progression within 1-year after initiation of androgen deprivation therapy. prostate-specific antigen response rate was 55.1% (95% confidence interval: 40.2%–69.3%), median prostate-specific antigen–progression-free survival was 24.1 weeks, and median overall survival was 102.9 weeks (95% confidence interval: 64.86 not estimable [NE]). Most common adverse event was nasopharyngitis (15/50 patients, 30.0%). The most common ≥grade 3 adverse event was alanine aminotransferase increased (6/50 patients, 12.0%).

**Conclusions:**

Abiraterone acetate plus prednisolone demonstrated a high prostate-specific antigen response rate of 55.1%, suggesting tumor growth still depends on androgen synthesis in patients with early metastatic castration-resistant prostate cancer. However, prostate-specific antigen–progression-free survival was shorter than that reported in previous studies. Considering the benefit–risk profile, abiraterone acetate plus prednisolone would be a beneficial treatment option for patients with chemotherapy-naive metastatic prostate cancer who show early castration resistance.

## Teaser

This study investigated efficacy and safety of abiraterone acetate plus prednisolone in chemotherapy-naive early metastatic castration-resistant prostate cancer patients. Abiraterone acetate plus prednisolone proved to be a beneficial treatment option with regards to its higher response rate.

## Introduction

Prostate cancer (PC) represents 7.1% of all new cancer cases and is the fifth leading cause of cancer-related deaths in men globally (1). According to the Cancer Statistics estimation in Japan, the number of PC incidence was 86 100, the incidence rate was ranked third among cancer in men in 2017, and the number of projected deaths were 12 200 in 2016 (sixth-leading site for cancer-related mortality in men) (2). For metastatic PC, castration is the standard-of-care for disease control. However, tumor progression occurs in most patients within a few years and the disease eventually progresses to metastatic castration-resistant PC (mCRPC). For mCRPC, several treatments were recommended including second-line hormonal therapy and chemotherapy (3).

The Japanese clinical guidelines for PC (2012) recommended antiandrogen therapy for the patients who previously responded to initial androgen deprivation therapy (ADT) (4). In patients receiving secondary hormonal therapy with conventional antiandrogen agents, the prostate-specific antigen (PSA) response rate ranged 30–50% [4–7]. In contrast, in patients with mCRPC with disease progression within 12 months of initial ADT or who had progression without achieving a normal PSA level (<4 ng/mL) during first-line ADT therapy, the PSA response rate was reported to be only 10% to 14% (5–7). These data suggest that patients who achieved early castration resistance to initial ADT are far more likely to exhibit an insufficient PSA response to conventional secondary hormonal therapy compared with others. Therefore, it was crucial to establish an effective strategy for the treatment of patients with mCRPC who responded insufficiently to initial ADT, however, no standard treatment options exist for this patient population.

Abiraterone acetate (AA) (Zytiga®), a prodrug of abiraterone, is a cytochrome P450 17 (CYP17) enzyme inhibitor that suppresses both the adrenal androgens pathway and de novo intra-tumoral androgen synthesis (8). In the COU-AA-302 study, treatment with AA plus prednisone/prednisolone improved overall survival (OS) (9) in patients with chemotherapy-naive mCRPC. The novel mechanism of action of AA suggests that patients with mCRPC who insufficiently responded to initial ADT could benefit with the AA plus prednisolone (P) (henceforth AAP) as a secondary hormonal therapy. Thus, in the current study, the efficacy and safety of AAP was investigated in patients with chemotherapy-naive mCRPC who experienced early onset of castration resistance (early mCRPC).

## Methods

This was a single-arm, non-randomized, two-stage design, multi-center (15 sites in Japan) study conducted in accordance with the ethical principles that have their origin in the Declaration of Helsinki and that are consistent with Good Clinical Practices and applicable regulatory requirements. The Institutional Review Board reviewed and approved the protocol, informed consent form and amendments. All patients provided written informed consent before enrollment. This study was registered at ClinicalTrials.gov (NCT02405858).

### Patient population

The primary eligibility criteria were: men aged ≥20 years with histologically or cytologically confirmed adenocarcinoma of the prostate who had metastatic disease and were, surgically or medically castrated, showed early castration resistance defined as PSA progression within a year after the start of first-line combined androgen blockade (CAB) therapy or who had PSA progression without having a normal PSA level (<4.0 ng/mL) in the first-line ADT (PSA progression should be confirmed by the Prostate Cancer Clinical Trials Working Group 2 criteria [PCWG2] criteria (10) after antiandrogen withdrawal), no history of chemotherapy with an Eastern Cooperative Oncology Group (ECOG) performance status score of 0–2, adequate hematologic function and ≤2.5 × upper limit of normal serum aspartate aminotransferase (AST) and alanine aminotransferase (ALT).

Patients with intolerance to ingredients of AAP or major medical condition including brain metastasis or local prostatic intervention within 4 weeks prior to AAP initiation were excluded.

### Treatment

Eligible patients were administered 1000 mg of AA orally (taken either ≥2 h before a meal or 1 h after a meal) and 10 mg of P, once a day. A 28 day-daily dosing cycle was continued until disease progression or unacceptable toxicity was observed. All patients who had not undergone orchiectomy were required to receive a luteinizing hormone-releasing hormone agonist or antagonist throughout the study to maintain castrate testosterone levels (<50 ng/dL or <1.7 nM). Maximally, two dose level reductions were allowed (one dose level was defined as 250 mg/day [one tablet of AA]) for adverse event (AE) management. A dose of AA <500 mg/day was not allowed and any dose increase was prohibited in this study.

### Outcomes

The primary endpoint was the proportion of patients achieving PSA response defined as the first occurrence of ≥50% PSA decline from baseline to 12 weeks according to PCWG2 criteria confirmed by a subsequent measurement at ≥4 weeks later.

Secondary endpoints included PSA-based progression-free survival (PSA–PFS), duration of PSA response, time to PSA response, radiographic (RAD)-PFS, OS, maximal serum PSA decline evaluation according to PCWG2 criteria and RAD-objective response rate (RAD-ORR) in patients with measurable lesions at baseline using response evaluation criteria in solid tumors (RECIST, version 1.1).

Safety evaluation were AEs, physical examinations, vital sign measurements and clinical laboratory tests (hematology, serum chemistry and liver function tests). AEs were collected from the start of study treatment until 30 days after discontinuation of study drugs with the causal relationship to study drugs. All AEs were evaluated according to the Common Terminology Criteria for Adverse Events (CTCAE) version 4.0.

### Statistical methods

The sample size was calculated according to Simon’s minimax design. Assuming an expected PSA response rate of 35% (i.e. 20% > the threshold response rate to 15% [5, 6]; of conventional antiandrogen replacement therapy), minimally 48 patients were required for efficacy analysis with α = 0.025 and 90% of statistical power. An interim analysis was set for efficacy evaluation; if the number of responders were ≤4 patients within the first 27 patients treated consecutively, further patient enrollment would be discontinued.

Efficacy was evaluated with patients who received ≥1 treatment with the study drugs and had any post-treatment PSA assessment data (efficacy analysis set). Safety was evaluated with patients who received ≥1 dose of study drugs (safety analysis set). All variables were summarized using descriptive statistics with two-sided exact 95% confidence interval (CI). Kaplan–Meier product-limit method was used to estimate the median time to event endpoints. All statistical analyses were performed using SAS version 9.3 (SAS Institute Inc., USA).

## Results

### Patient disposition, characteristics and treatment exposure

Total 50 patients were enrolled from May 2015 to January 2017. The median age was 73 (range: 55–86) years. At initial diagnosis, the median PSA level was 433.35 (range: 7.36–13740.12 ng/mL) and 90.0% of the patients showed ≥8 total Gleason Score and score of 9 was most common (31 patients, 62.0%). The median PSA levels before initiating AAP was 27.47 (range: 2.281–294.245) ng/mL. The median duration of initial ADT was 32.4 (range: 13.4–84.1) weeks and 48 patients experienced PSA progression within one year after initiation of ADT (Table 1). All 50 patients received first dose of study drugs, one patient was excluded because of consent withdrawal before post-treatment PSA assessment. All 50 patients were included in the safety analysis. The median cycles of treatment were 8.86 (range: 0.5–27.9). The most common reason for treatment discontinuation was physician’s decision (included requirements for other anti-tumor therapy due to unequivocal clinical progression) (22 patients, 44.0%), followed by progressive disease (15 patients, 30.0%). Of the 50 patients, 34 (68.0%) received subsequent therapies; docetaxel (23 [46.0%]), enzalutamide (12 [24.0%]) AA (9 [18.0%]) and radiotherapy to bone. (8 [16.0%]) were the most frequent.

**Table 1:** Demographics and baseline characteristics (safety analysis set)

**Characteristics**	*n* = 50
**Age** (years), median (range)	73 (55–86)
**Body weight** (kg), mean (SD)	65.52 (11.22)
**Serum PSA** ^**a**^ (ng/mL), median (range)	433.35 (7.36–13740.12)
**Total Gleason Score** ^**a**^	
6	1 (2.0)
7	1 (2.0)
8	5 (10.0)
9	31 (62.0)
10	9 (18.0)
Unknown	3 (6.0)
**Extent of disease** ^**b**^	
Bone	44 (88.0)
Prostate mass	21 (42.0)
Lymph node	20 (40.0)
Bladder	4 (8.0)
Lungs	4 (8.0)
Seminal vesicles	2 (4.0)
**ECOG performance status**	
Category, *n*	
0	40
1	8
2	2
**Previous prostate cancer therapy**	
Radiotherapy	2 (4)
Hormone	
Bicalutamide	50 (100)
**Orchiectomy**	7 (14)
**Duration of initial ADT** (weeks), median (range)	32.36 (13.40–84.10)
**PSA progression after initiation of ADT**	
≤52 weeks	48 (96.0)
>52 weeks	2 (4.0)

^a^At initial diagnosis.

^b^Multiple count.

Values are presented as *n* (%) unless specified.

Abbreviations: ADT: androgen deprivation therapy; ECOG: Eastern Cooperative Oncology Group; n: number of patients; PSA: prostate-specific antigen; SD: standard deviation.

### Efficacy

**Figure 1.**
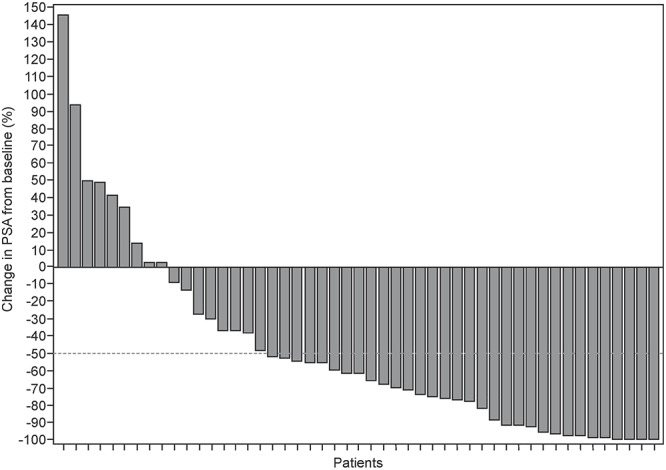
Waterfall plot: Maximum percent change of serum prostate-specific antigen (PSA) level from baseline (full analysis set).

**Figure 2.**
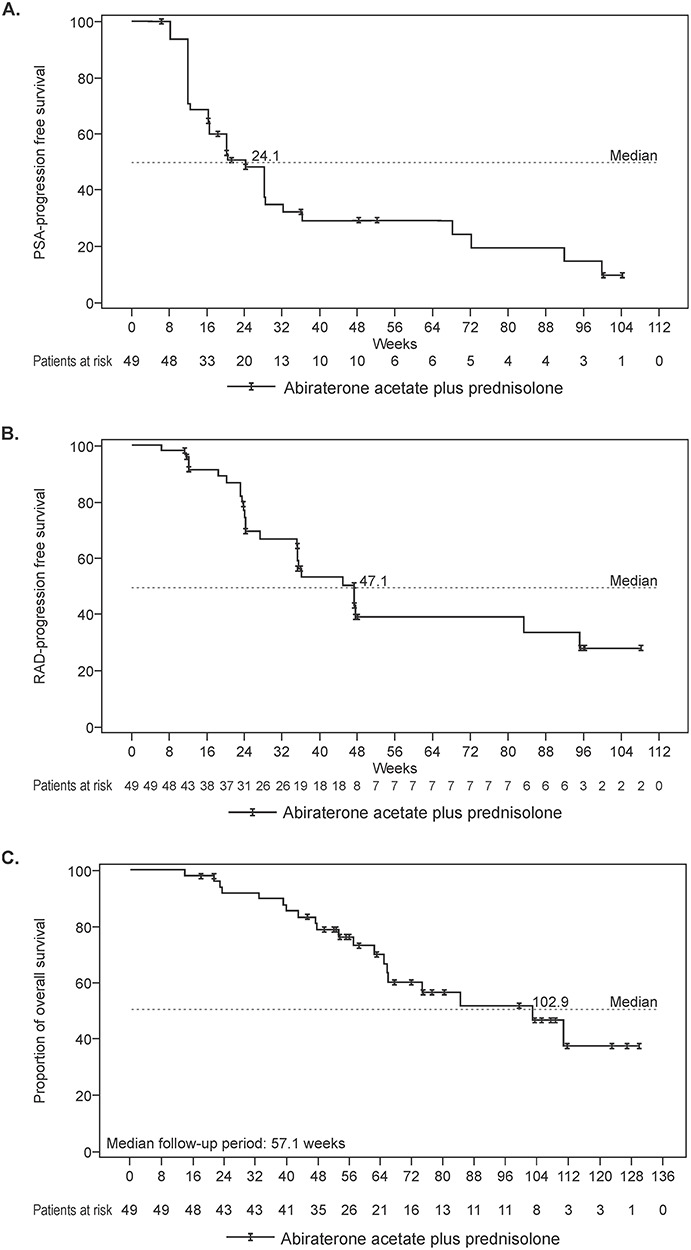
Kaplan–Meier plot (A) PSA-progression free survival, (B) radiographic-progression free survival and (C) overall survival (full analysis set).

Of the 49 patients, 27 achieved PSA response and the PSA response rate was 55.1% (95% CI: 40.2%–69.3%) by week 12. The lower limit of the two-sided 95% CI (40.2%) exceeded the predefined threshold value of 15%. Figure 1 shows the waterfall plot of maximal change of PSA from baseline, the median percent change in PSA level from baseline was 61.9% (range: –100.0% to 146.0%). The median time to PSA response was 28.5 (95% CI: 28.0–56.0) days and the median duration of PSA response was 24.1 (95% CI: 12.0–64.0) weeks in these 27 patients. The median PSA–PFS was 24.1 (95% CI: 16.14–28.29) weeks (Fig. 2A). The median RAD–PFS from the first dose of study drug for 49 patients was 47.1 (95% CI: 35.00–95.00) weeks (Fig. 2B).

Measurable lesions were present in 9 (18.37%) patients at baseline and of these 9 patients, 1 patient each achieved CR and PR and met the criteria for RAD–ORR, thus RAD–ORR was 22.2% (95% CI: 2.8–60.0%). With the median follow-up period of 57.1 weeks, the median OS from the first dose of study drugs was 102.9 weeks (95% CI: 64.86 not estimable) (Fig. 2C).

### Safety

AEs of worst severity that occurred in >5% of patients are presented in Table 2. The most common (in ≥10% patients) AE was nasopharyngitis (15/50 patients, 30.0%), followed by ALT increased (14/50 patients, 28.0%). The most common (in ≥10.0% patients) grade 3 AEs were ALT increased (6/50 patients, 12.0%) followed by AST increased and hyperglycemia. In AAP treatment group, of the patients with grade 3 liver dysfunction, 1 patient needed dose reductions and 4 patients needed interruptions due AEs. No dose adjustment was performed as the one patient who developed grade 3 ALT increase displayed a transient elevation.

**Table 2:** Summary of adverse events (in >5% patients, safety analysis set)

Graded AEs^a^	*n* = 50, *n* (%)			
	Grade 1	Grade 2	Grade 3	Total
Nasopharyngitis	14 (28.0)	1 (2.0)	0	15 (30.0)
Alanine aminotransferase increased	4 (8.0)	4 (8.0)	6 (12.0)	14 (28.0)
Aspartate aminotransferase increased	5 (10.0)	3 (6.0)	5 (10.0)	13 (26.0)
Alkaline phosphatase increased	6 (12.0)	1 (2.0)	3 (6.0)	10 (20.0)
Blood lactate dehydrogenase increased	9 (18.0)	0	0	9 (18.0)
Back pain	4 (8.0)	2 (4.0)	0	6 (12.0)
Hyperglycemia	1 (2.0)	0	5 (10.0)	6 (12.0)
Constipation	5 (10.0)	1 (2.0)	0	6 (12.0)
Malaise	5 (10.0)	0	0	5 (10.0)
Weight decreased	5 (10.0)	0	0	5 (10.0)
Vomiting	4 (8.0)	0	0	4 (8.0)
Gamma-glutamyl transferase increased	2 (4.0)	2 (4.0)	0	4 (8.0)
Diabetes mellitus	1 (2.0)	3 (6.0)	0	4 (8.0)
Hypertension	0	0	2 (4.0)	2 (4.0)
Abdominal pain upper	3 (6.0)	0	0	3 (6.0)
Diarrhea	1 (2.0)	1 (2.0)	1 (2.0)	3 (6.0)
Nausea	1 (2.0)	2 (4.0)	0	3 (6.0)
Osteonecrosis of jaw	2 (4.0)	1 (2.0)	0	3 (6.0)
Oedema peripheral	2 (4.0)	1 (2.0)	0	3 (6.0)
Pyrexia	3 (6.0)	0	0	3 (6.0)

^a^In >5% patients. No grade 4/5 AEs were observed. Abbreviations: AEs: adverse events.

Serious AEs (SAEs) reported in this study were the atrial flutter, hypercalcemia, cancer pain, chronic obstructive pulmonary disease, bone marrow failure, gastric ulcer and lung malignant neoplasm (1 patient each, 2.0%). Of these SAEs, the causal relationship to either of the study drugs was reported in atrial flutter, hypercalcemia and gastric ulcer by investigators.

## Discussion

This is a pioneering prospective study evaluating the efficacy and safety of AAP treatment in patients with chemotherapy-naïve early mCRPC. The PSA response rate after AAP was 55.1% treatment by week 12, which was ≥40.2% than the predefined threshold set by traditional antiandrogen alternating therapy.

Approximately 20% of patients with metastatic or recurrent PC progressed within 1-year after the initiation of first-line ADT (11). In this patient population, an insufficient PSA response to secondary hormonal therapy and a shorter duration of response and OS has been observed in a few retrospective studies (5, 6, 12). Prior to approval of docetaxel, some drugs including flutamide were recommended to treat patients with PC in Japan; however, the response was inadequate (7, 12). Later, docetaxel was approved for patients with early mCRPC and PSA response rate of 50% was reported in retrospective studies (13, 14). The maximal PSA decline observed in the present study (61.9%), was comparable with the COU-AA-302 study (62.0%) of AAP conducted in similar settings (8). As AA is considered to block both the adrenal androgens pathway and de novo intra-tumoral androgen synthesis (8), observed PSA response rate in the present study indicated that most of the patients with early CRPC still possess androgen-dependence.

Despite satisfactory PSA response rate observed in the present study, duration of response was shorter than that reported in the previous study (8). The median PSA–PFS and RAD–PFS were 5 months shorter in the current study than reported in the COU-AA-302 study (8). In an exploratory analysis of the COU-AA-302 study, longer prior exposure to endocrine therapy impacted RAD–PFS of AAP in patients with chemotherapy-naive mCRPC (15). Similar to this study, shorter duration of efficacy was also reported in retrospective studies including docetaxel and enzalutamide (12–14, 16). Thus, this study confirmed that patients with early mCRPC are considered to acquire relatively precocious drug resistance in comparison to the patients with non-early mCRPC. Several mechanisms for acquisition of CRPC have been proposed, however, an exact mechanism is still not inferred (17). Therefore, it is difficult to attempt to improve the outcome for these patients based on molecular biology considerations. In this study, 90% of patients exhibited relatively higher Gleason score of ≥8. Considering the subset analysis of COU-AA-302 study, high Gleason score might be related to shorter duration of efficacy of AA plus prednisolone/prednisone (18). Nevertheless, one-quarter of patients were PSA-progression free in this study for >1 year from the start of treatment. Therefore, further studies are warranted to evaluate the predictive factors for the effectiveness of AAP including the PSA response and PSA–PFS for patients with early CRPC.

Safety profile of AAP in this study was comparable with the COU-AA-302 study, except liver transaminase elevations (8). However, the safety profile was consistent with the phase 2 study in Japanese patients with mCRPC treated with AAP (19, 20). Since no new safety signals were identified in the current study, AAP was tolerable for this patient population as well. This open-label non-randomized study has limitations inherent to the study design.

Presently, there is no consensus for the treatment of patients with early mCRPC, as established data are not available for the therapeutic strategies that might improve the OS of these patients (21, 22). Docetaxel is recommended for the treatment of patients with early mCRPC based on the findings from retrospective studies using docetaxel in this patient population (23); however, management of AEs is critical due to its cytotoxic nature. Collectively, these findings indicate that an alternative treatment option incorporating second-line hormonal therapy, such as AAP, would be beneficial for these patients.

## Conclusion

In this study, AAP demonstrated a higher PSA response rate than the predefined response rate. The result suggests that tumor growth still depends on androgen synthesis even in patients who exhibit early castration resistance. Although duration of effectiveness was relatively shorter, considering the benefit–risk profile, AAP would be one of the treatment options for patients with chemotherapy-naive early mCRPC.

## Author contributions

Kazuki Kobayashi, Norihiko Okuno, Gaku Arai, Hiroomi Nakatsu, Akimitsu Maniwa, Naoto Kamiya, Takefumi Satoh, Hiroaki Kikukawa, Yoshitsugu Nasu, Hiroji Uemura, Takao Nakashima, Kazuo Mikami, Masahiro Iinuma, Kazunari Tanabe and Junya Furukawa were the investigators, and Hisanori Kobayashi was the project statistician. Tomihiro Takahara, Mizue Ogi and Masahiko Nakayama had primary roles in the study design, results assessment and data interpretation as clinical lead for the study. All authors contributed to the data interpretation for the results.

All authors met ICMJE criteria and all those who fulfilled those criteria are listed as authors. All authors had access to the study data and made the final decision about where to publish these data and approved submission to this journal.

## Funding

This work was supported by funding from Janssen Pharmaceutical K.K., Tokyo, Japan.

## Conflict of interest statement

Dr Takefumi Satoh received grants from Konica Minolta, Inc.; consultation fee from Janssen Pharmaceutical K.K.; and educational lecture fee from Bayer AG, AstraZeneca, Janssen Pharmaceutical K.K., Astellas Pharmaceutical Inc., Nihon Medi-Physics Company Limited and Takeda Pharmaceutical Company Limited. Dr Hiroji Uemura received honoraria from Janssen Pharmaceutical K.K., Sanofi, Astellas Pharmaceutical Inc. and Bayer AG; consultant and advisor for Janssen, Bayer AG; in speaker’s bureau for Janssen Pharmaceutical K.K., Bayer AG; received a travel grant from Bayer AG and MSD.

The following author declare a conflict of interest on the basis that he is a full-time employee of Janssen Pharmaceutical K.K. of Johnson & Johnson: Hisanori Kobayashi. All remaining authors have declared no conflicts of interest. The material presented in this paper reflects authors own personal views and should not be interpreted as being representative of the views of their employers or institutions.

## References

[1] Bray F, Ferlay J, Soerjomataram I, Siegel RL, Torre LA, Jemal A. Global cancer statistics 2018: GLOBOCAN estimates of incidence and mortality worldwide for 36 cancers in 185 countries. CA Cancer J Clin 2018;68:394–424. 30207593 10.3322/caac.21492

[2] CANCER STATISTICS IN JAPAN ’17. [Cancer information Service, National Cancer Center Japan]. Ganjoho.jp. https://ganjoho.jp/en/professional/statistics/brochure/2017_en.html (18 November 2020, date last accessed).

[3] Horwich A, Parker C, Bangma C, Kataja V, Group EGW. Prostate cancer: ESMO clinical practice guidelines for diagnosis, treatment and follow-up. Ann Oncol 2010;21:v129–33. 20555062 10.1093/annonc/mdq174

[4] Kakehi Y, Sugimoto M, Taoka R. Association tcfeote-bcpgfpcotJU. Evidenced-based clinical practice guideline for prostate cancer (summary: Japanese Urological Association, 2016 edition). Int J Urol 2017;24:648–66. 28667698 10.1111/iju.13380

[5] Miyake H, Hara I, Eto H. Clinical outcome of maximum androgen blockade using flutamide as second-line hormonal therapy for hormone-refractory prostate cancer. BJU Int 2005;96:791–5. 16153202 10.1111/j.1464-410X.2005.05766.x

[6] Suzuki H, Okihara K, Miyake H, et al. Alternative nonsteroidal antiandrogen therapy for advanced prostate cancer that relapsed after initial maximum androgen blockade. J Urol 2008;180:921–7. 18635218 10.1016/j.juro.2008.05.045

[7] Okegawa T, Nutahara K, Higashihara E. Alternative antiandrogen therapy in patients with castration-resistant prostate cancer: a single-center experience. Int J Urol 2010;17:950–5. 20807265 10.1111/j.1442-2042.2010.02620.x

[8] Ryan CJ, Smith MR, de Bono JS, et al. Abiraterone in metastatic prostate cancer without previous chemotherapy. N Engl J Med 2013;368:138–48. 23228172 10.1056/NEJMoa1209096 PMC3683570

[9] Ryan CJ, Smith MR, Fizazi K, et al. Abiraterone acetate plus prednisone versus placebo plus prednisone in chemotherapy-naive men with metastatic castration-resistant prostate cancer (COU-AA-302): final overall survival analysis of a randomised, double-blind, placebo-controlled phase 3 study. Lancet Oncol 2015;16:152–60. 25601341 10.1016/S1470-2045(14)71205-7

[10] Scher HI, Halabi S, Tannock I, et al. Design and end points of clinical trials for patients with progressive prostate cancer and castrate levels of testosterone: recommendations of the prostate cancer clinical trials working group. J Clin Oncol 2008;26:1148–59. 18309951 10.1200/JCO.2007.12.4487 PMC4010133

[11] James ND, Sydes MR, Clarke NW, et al. Addition of docetaxel, zoledronic acid, or both to first-line long-term hormone therapy in prostate cancer (STAMPEDE): survival results from an adaptive, multiarm, multistage, platform randomised controlled trial. Lancet (London England) 2016;387:1163–77. 10.1016/S0140-6736(15)01037-5 PMC4800035 26719232

[12] Bournakis E, Efstathiou E, Varkaris A, et al. Time to castration resistance is an independent predictor of castration-resistant prostate cancer survival. Anticancer Res 2011;31:1475–82. 21508406

[13] Huillard O, Albiges L, Eymard J-C, et al. Efficacy of docetaxel chemotherapy in metastatic prostate cancer (mPC) patients (pts) experiencing early castration resistance (CR). J Clin Oncol 2013;31:5075.

[14] Angelergues A, Maillet D, Flechon A, et al. Duration of response to androgen-deprivation therapy (ADT) and efficacy of secondary hormone therapy, docetaxel (D), and cabazitaxel (C) in metastatic castration-resistant prostate cancer (mCRPC). J Clin Oncol 2014;32:282.24220559

[15] Oudard S, Kheoh TS, Yu MK, et al. Impact of prior endocrine therapy on radiographic progression-free survival (rPFS) in patients (pts) with chemotherapy-naive metastatic castration-resistant prostate cancer (mCRPC): results from COU-AA-302. J Clin Oncol 2014;32:14.

[16] Loriot Y, Eymard JC, Patrikidou A, et al. Prior long response to androgen deprivation predicts response to next-generation androgen receptor axis targeted drugs in castration resistant prostate cancer. Eur J Cancer (Oxford England: 1990) 2015;51:1946–52. 10.1016/j.ejca.2015.06.128 26208462

[17] Tsao CK, Galsky MD, Small AC, Yee T, Oh WK. Targeting the androgen receptor signalling axis in castration-resistant prostate cancer (CRPC). BJU Int 2012;110:1580–8. 22985411 10.1111/j.1464-410X.2012.11445.x

[18] Fizazi K, Flaig TW, Stöckle M, et al. Does Gleason score at initial diagnosis predict efficacy of abiraterone acetate therapy in patients with metastatic castration-resistant prostate cancer? An analysis of abiraterone acetate phase III trials. Ann Oncol 2016;27:699–705. 26609008 10.1093/annonc/mdv545 PMC6279107

[19] Satoh T, Uemura H, Tanabe K, et al. A phase 2 study of abiraterone acetate in Japanese men with metastatic castration-resistant prostate cancer who had received docetaxel-based chemotherapy. Jpn J Clin Oncol 2014;44:1206–15. 25425730 10.1093/jjco/hyu148 PMC4243578

[20] Matsubara N, Uemura H, Satoh T, et al. A phase 2 trial of abiraterone acetate in Japanese men with metastatic castration-resistant prostate cancer and without prior chemotherapy (JPN-201 study). Jpn J Clin Oncol 2014;44:1216–26. 25320340 10.1093/jjco/hyu149 PMC4243579

[21] Heidenreich A, Bastian PJ, Bellmunt J, et al. EAU guidelines on prostate cancer. Part II: treatment of advanced, relapsing, and castration-resistant prostate cancer. Eur Urol 2014;65:467–79. 24321502 10.1016/j.eururo.2013.11.002

[22] Virgo KS, Rumble RB, Singer EA. Second-line hormonal therapy for men with chemotherapy-Naïve castration-resistant prostate cancer: American Society of Clinical Oncology provisional clinical opinion summary. J Oncol Pract 2017;13:459–61. 28445101 10.1200/JOP.2017.022970

[23] Lorente D, Fizazi K, Sweeney C, de Bono JS. Optimal treatment sequence for metastatic castration-resistant prostate cancer. Eur Urol Focus 2016;2:488–98. 28723514 10.1016/j.euf.2016.10.008

